# Ammonia, or Hartshorn

**Published:** 1884-08

**Authors:** 


### AMMONIA ; OR, HARTSHORN.

THE original source of supply of ammonia was camel's dung. It is found in the refuse matters of men and animals, and especially in the urine. It is this that produces the pungent and sickening odor in urinals and neglected stables. It is a corrosive poison in its concentrated form, and peculiarly destructive to the delicate tissues of the animal economy. The action of ammonia on the sensitive membranes of the eye is the most prominent cause of blindness in horses. Animals that are confined in close stables, where manure is allowed to accumulate in considerable quantity, are, in addition to blindness, peculiarly liable to diseases of the lungs and the kidneys, from the action of ammonia. The effect of this drug seems to be cumulative, and when taken into the stomach in small doses repeatedly it is easy to conceive that its action must be irritating to the coating of the stomach, and to those far more sensitive membranes that line the passage of the urinary apparatus.

Ammonia is largely used in the manufacture of baking powders, and probably more than one-half the cans of baking powder on every grocer's shelves contain it. Fortunately the test is very simple for this material, which is in no sense a food, and which should never be permitted to get into the stomach except by the advice of a competent physician. Whenever a can of baking powder is purchased, no matter what the "brand" may be, the safety of the family requires that it should be tested. All that is necessary to make the test is to place the can, cover down, on a hot stove for a minute or two ; then take off the cover and smell. If you get the pungent fumes of ammonia, discard the baking powder and avoid the cheat in futnre. There are plenty of baking powders in the market that are made ONLY from pure cream-of-tartar and bicarbonate of soda, with a little flour or starch combined as a preservative. Buy only baking powders that have printed guarantees on the labels, or in the circulars contained in the cans. The word "pure" has been prostituted to base uses. It has no meaning in the sense in which it is often used, and it is made to cover the vilest compounds. Oleomargarine may be absolutely pure, but it is not butter. No honest dealer in any class of goods intended as food, or to enter into foods, will refuse to furnish with each package the correct formula from which the goods are made. Every consumer has a right to know what he is using as food. We always refuse to buy goods of any kind that are branded PURE, STRICTLY PURE, or ABSOLUTELY PURE, unless in addition we are as plainly informed of just what they are made ; and we think this rule is always a safe one.

Although ammonia is not a food, it has its uses. It is one of the best of remedies as an application in bites of dogs and serpents, and the stinging of bees and other insects. When promptly applied it destroys the poison, and also the tissue which has been impregnated with the poison, very much as a red-hot iron would do the same thing. Ammonia is used in smelling-bottles, for headache ; it gets up a counter-irritation in the nasal passages that tends to draw the pain from where it was located. Ammonia is also much used for removing grease-spots from garments. By its caustic action it converts the grease into soap, which can be washed out with water. It should be kept beyond the reach of children, if it is to be kept on hand at all, as fatal accidents have occurred to children and others who have used it carelessly and in ignorance of its dangerous properties.

